# A Study Investigating Whether BMI Is Associated With Acetabular Bone Size: Big Bones or a Big Myth?

**DOI:** 10.7759/cureus.19766

**Published:** 2021-11-20

**Authors:** Jaison Patel, George Hourston, Stephen M McDonnell

**Affiliations:** 1 Department of Trauma and Orthopaedic Surgery, Addenbrooke's Hospital, Cambridge University Hospitals NHS Foundation Trust, Cambridge, GBR; 2 Department of Trauma and Orthopaedics, James Paget University Hospitals NHS Foundation Trust, Great Yarmouth, GBR

**Keywords:** big bones, acetabular cup, hip replacement, hip arthroplasty, obesity, implant size, bone morphology, bone size

## Abstract

Background

There is a common conception held by patients with a high body mass index (BMI) that they have “big bones”. Some people hold the assumption that their weight is attributed to larger bone stock rather than adipose tissue. It was the suspicion of the surgeons at our unit that this is often not the case. We therefore conducted a study investigating if there is any association between BMI and acetabular bone size.

Methods

We conducted a retrospective chart review of all patients undergoing total hip arthroplasty using the Trident acetabular system from Stryker at our tertiary level 1 trauma centre between September 2016 and August 2020. Patient demographic and surgical data were collected, and the association of BMI, height, and weight, with acetabular cup size was investigated using Pearson’s correlation coefficient and chi-square test for independence.

Results

A total of 418 patients were included in this study (52.4% female; age: 20-93 years; mean age: 62.51 years), with a mean BMI of 29.55 kg/m^2^(range: 14.95-52.32 kg/m^2^). A weak positive association between BMI and cup size, which was statistically significant (r = 0.107; n = 418; p = 0.02). The chi-square test for independence was used to study the association between obesity and cup size (large vs small), which demonstrated no significant difference (p = 0.08). There was a moderately strong positive association between height and cup size (r = 0.551; n = 418; p < 0.01). There was a weak positive association between weight and cup size, which was statistically significant (r = 0.355; n = 418; p < 0.01).

Conclusion

Our study suggests that there is indeed a weakly positive linear association between BMI and cup size among total hip arthroplasty patients. This effect was, however, more significant for height and weight, and there was no significant association between obese and non-obese groups with small versus large cup size implanted. We therefore conclude that clinically there is no significant relationship between obesity and acetabular bone size and that the “big bones” claim is indeed fallacious.

## Introduction

Obesity and poor bone health are significant and worsening causes of morbidity and mortality globally [1-3]. There has been a great deal of research into the interplay between increased body mass index (BMI) and fracture risk [4]. This relationship appears to be complex, with some protective effect of increased body mass on bone density; however, the higher the BMI, the lower this protective effect seems to be. Anecdotally, it is often claimed that there is an association between increased BMI and bone “size”, although there is minimal evidence in the literature substantiating this idea. Indeed, skeletal micro- and macrostructure development is challenging to investigate among children in different weight categories because obese children tend to be more physiologically and skeletally mature than their healthy counterparts [5]. There is a scarcity of data for or against the idea that overweight individuals have big bones. We therefore sought to investigate whether any association exists between obesity and skeletal macrostructure using acetabular cup size in order to begin to validate or disprove this claim.

We decided to collect data for patients undergoing total hip replacement (THR) to address this question. In THR surgery, there is little variation in the desired cup position and surgical technique when inserting uncemented acetabular components. When reaming the acetabulum, the surgeon aims to insert the component in approximately 45° abduction and 20° anteversion. Depending on the surgical technique recommended by the manufacturer, the patient’s acetabulum is reamed medially to the true floor and circumferentially 1-2mm below the desired shell size. The variation therefore lies in the circumferential size of the shell which relates to the size of the native pelvis. Further studies have shown that there is minimal variation in the cup size templated by surgeons in planning for total hip arthroplasty, lending this method to be an accurate one to have the potential to assess the bone size of a large population of patients [6,7]. Thus, data on the cup size used intra-operatively can be used as a surrogate for skeletal macro-architecture. Our null hypothesis states that there is no association between BMI and acetabular cup size in THR.

## Materials and methods

We performed a retrospective chart review identifying all patients who had undergone a THR using the Trident system and having a Hemispherical shell© (Stryker Corporation, Kalamazoo, MI) inserted in our centre, a level 1 major trauma centre in the United Kingdom, between September 8, 2016, and August 3, 2020. The project was approved by the local audit and research department (ref 326639). Data were collected using the hospital electronic patient record system EPIC (Epic Systems Corporation, United States). Criteria for inclusion included uncomplicated THRs in all patients, whereas exclusion criteria included revision arthroplasty and complex primary arthroplasty. Complex hip arthroplasty was defined as any procedure in which a patient had non-standard hip prosthesis use, bone grafting, post-traumatic arthritis, protrusio, or skeletal dysplasia. Demographic data including patient age, gender, height and weight were collected, as well as surgical data such as acetabular shell size and senior surgeon responsible. The underlying primary pathology of the hip was also noted. Surgeons included in this study undreamed the pelvis by 1-2mm as per the surgical technique indicated by Stryker and varied depending on the fit achieved in trialling. Surgeons reamed the acetabulum to the true floor and expanded the cup up to the point at which the reamer engaged the superior portion of the acetabulum, hence creating a hemisphere.

Statistical analysis was performed using SPSS for Mac Version 26 (SPSS Inc., Chicago, IL). Pearson’s correlation coefficient was used to identify any associations between the normally distributed data for the collected variables, namely BMI (kg/m^2^), height (m), weight (kg) and age. The BMI was grouped into obese (BMI 30 and above) and not obese (BMI less than 30), and cup size was grouped into small (size 52mm and smaller) and large (size 54mm and larger). A chi-square test for independence was performed based on these groups. A p-value of less than 0.05 was deemed statistically significant.

## Results

A total of 462 patients were identified through our search. Once exclusion criteria were applied, a total of 418 patients were used for this study. The study excluded 11 patients who had the use of bone substitute, 16 patients who underwent complex primary hip arthroplasty and 15 patients who underwent revision total hip arthroplasty. Patient demographic data are demonstrated in Table 1. Females were more common in our cohort (52.4%; n = 219). The most frequently used cups were 50mm and 54mm (range = 44-64 mm), and most procedures were performed by a surgeon (n = 126). The underlying pathology leading to total hip arthroplasty were assessed: primary osteoarthritis (n = 290), developmental dysplasia of the hip (n = 89), inflammatory arthritis (n = 29) and post-infection (n = 10).

**Table 1:** Patient demographics

	Age	Height (m)	Weight (kg)	BMI (kg/m^2^)
Mean	62.51	1.67	83.14	29.55
Median	65.00	1.68	81.30	28.59
Standard deviation	12.65	0.10	20.76	6.54
Range	73.00	0.77	125.60	37.37
Minimum	20.00	1.23	40.70	14.95
Maximum	93.00	2.00	166.30	52.32

Correlation between BMI and cup size

**Figure 1:**
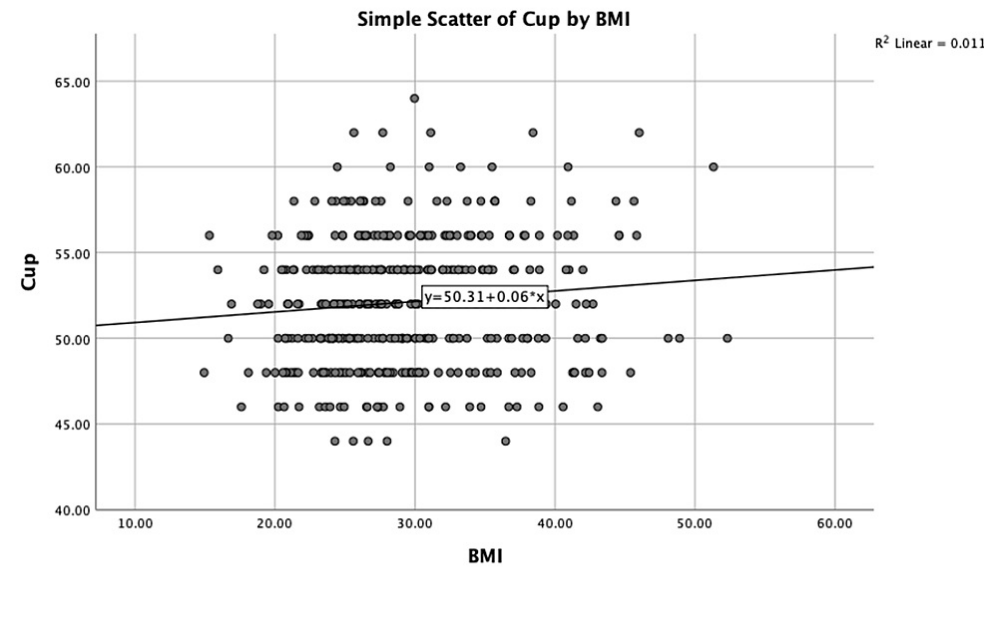
Scatter plot showing the relationship between BMI (kg/m2) and cup size (mm)

A Pearson product-moment correlation was performed to determine the relationship between BMI and cup size. There was a weak positive association between BMI and cup size, which was statistically significant (r = 0.107; n = 418; p = 0.02). Partial Pearson’s correlations whilst controlling for age (r = 0.108; p = 0.03) and surgeon (r = 0.118; p = 0.02.) were also performed. A scatter chart with fit line at total (R^2^ = 0.01) is shown in Figure 1.

**Figure 2:**
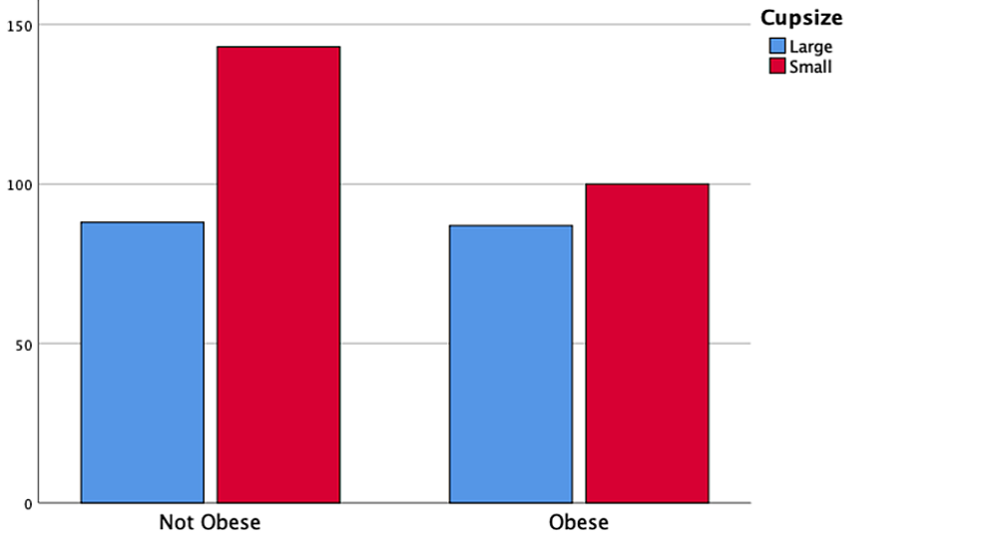
Bar chart showing cup sizes used in obese and not-obese groups

A chi-square test for independence was run based on the groups: obese (n = 187) and not obese (n = 231) with small cup and large cup. The relation between BMI and cup size was not significant (p = 0.08). The bar chart in Figure 2 demonstrates an equal number of large cups being used in the obese and not-obese groups (n = 87).

Correlation between height, weight and cup size

**Figure 3:**
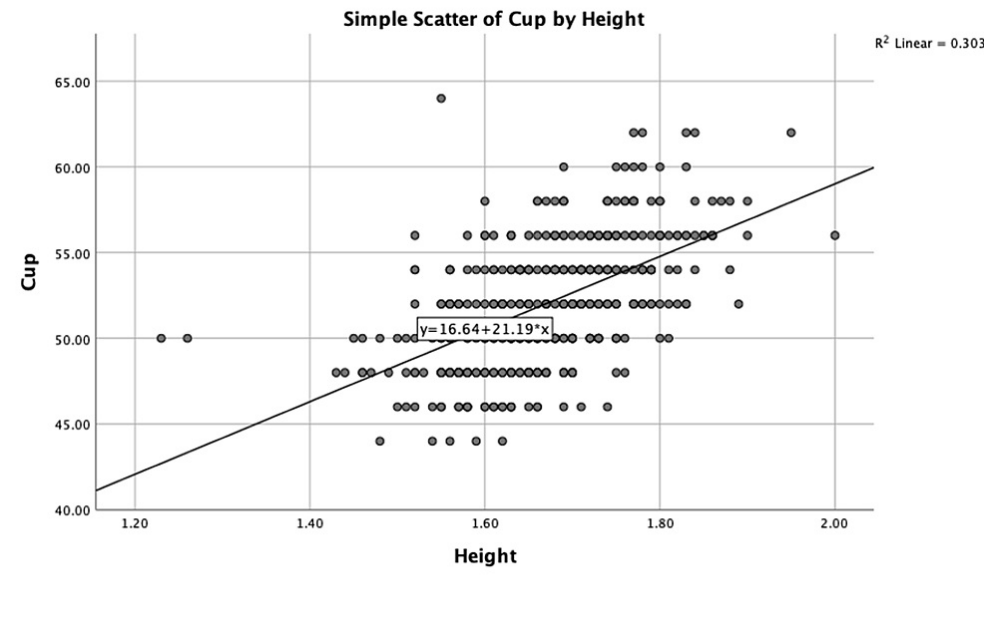
Scatter plot showing the relationship between height (m) and cup size (mm)

A Pearson product-moment correlation was performed to determine the relationship between height and cup size and between weight and cup size. There was a moderately strong positive association between height and cup size, which was statistically significant (r = 0.551; n = 418; p < 0.01.) Partial Pearson’s correlations whilst controlling for age (r = 0.589; p < 0.01) and surgeon (r = 0.547; p < 0.01.) were run. A scatter chart with fit line at total (R^2^ = 0.303) is shown in Figure 3.

**Figure 4:**
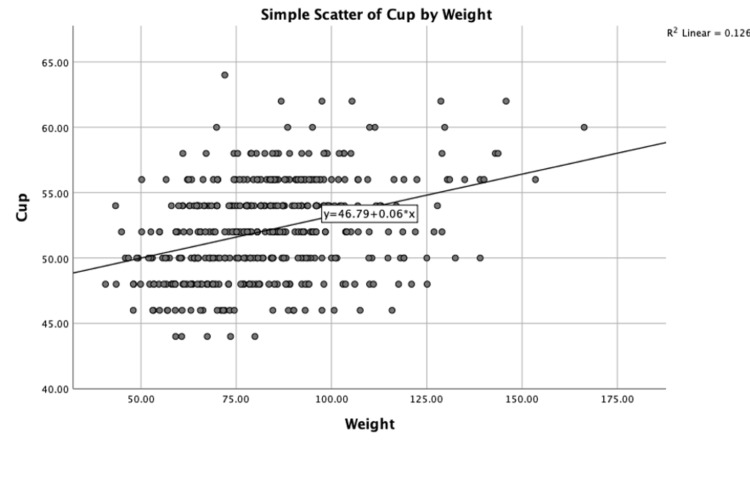
Scatter plot showing the relationship between weight (kg) and cup size (mm)

There was a weak positive association between weight and cup size, which was statistically significant (r = 0.355; n = 418; p < 0.01) Partial Pearson’s correlations whilst controlling for age (r = 0.372; p < 0.01) and surgeon (r = 0.362; p < 0.01) were run. A scatter chart with fit line at total (R^2^ = 0.126) is shown in Figure 4.

Correlation between gender and cup size

**Figure 5:**
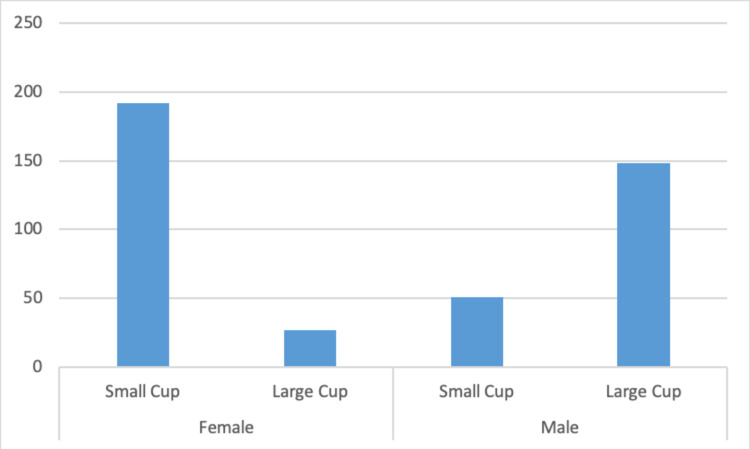
Number of small and large cup sizes used in male and female patients

A chi-square test for independence was run based on the group’s male (n = 199) and female (n = 219) with small cup and large cup. The relation between gender and cup size was significant (p < 0.01). The bar chart in Figure 5 shows the distribution of cup size by gender. In the female group, the mean age, height, weight and BMI were 62.3 years (range: 20-93 years), 1.62m (range: 1.23-1.83m), 76.2kg (range: 40.7-139.0kg) and 29.1 (range: 14.9-52.3), respectively. In the male group, the mean age, height, weight and BMI were 62.8 years (range: 20-89 years), 1.7m (range: 1.55-2.00m), 90.8kg (range: 49.3-166.3kg) and 30.1 (range: 15.3-51.4), respectively.

## Discussion

This study is unique in describing the relationship between obesity and acetabular bone size, using acetabular cup size as a surrogate for bone size. Although a positive correlation was found between BMI and cup size, it was weak. The positive correlation between weight and cup size was fair. A moderate positive correlation was found between height and cup size, which was the strongest correlation found in our study. A correlation was found with regards to gender, with female patients more likely to require a smaller sized acetabular cup and male patients to require larger cup sizes. However, the patient’s age, gender and surgeon did not influence the strength of the correlation as the patient’s BMI was independent of these factors. The relationship between gender and cup size is likely to be related to the mean height differences found between males and females (1.74m and 1.62m, respectively). The mean BMI between genders did not differ significantly. No positive correlation was found between the obese and non-obese patients and the size of the acetabular cup that was implanted during THR surgery. This study proves that the claim to be “big boned” is an unsupported one and that obese patients are just normal bone sized and this does not affect the acetabular cup size implanted.

Obesity has become a major world health burden. It is increasingly common among the orthopaedic patient population. Obesity is well known to influence hip arthroplasty, with higher rates of complications including wound complications, dislocation and length of stay, as well as increased risk of readmission and mortality [6]. Foot and ankle, knee surgery, shoulder and elbow and spine surgery are also affected in a similar way [7-13]. Understanding the effect of obesity on any given procedure and the potential increased risk it poses is crucially important for orthopaedic surgeons.

Owing to the well-documented increased risk that patients with a high BMI face, these patients may be urged away from surgical interventions. In 2016, 98 of 209 clinical commissioning groups (CCG) in the UK had some form of BMI threshold to funding of hip and knee arthroplasty [14]. This is done in the best interests of patients and with consideration given to healthcare economics due to the risks and poorer outcomes that have been well described in the literature. The authors of the papers agree that this restriction should not prevent patients with a high BMI who will have a clear benefit from arthroplasty to undergo surgery. However, patients may fail to understand this and feel let down or discriminated against by their surgeon. Weight loss prior to surgery has been shown to improve outcomes and reduce the complication profile faced by these patients [15-17]. As a result, it is highly recommended that patients attempt to lose weight prior to undergoing some surgical procedures. Patients may argue that weight loss is not possible because their weight contribution is largely attributed to their bone size (“big boned”). This study helps provide surgeons with evidence that the patients’ BMI does not correlate strongly with their bone size and therefore weight loss is indeed possible and should be attempted owing to the significant benefit is poses.

Predicting implant sizes is important when considering implant inventory. The ability to better predict sizes of implants can help institutions reduce costs and prevent waste. Although templating provides surgeons with the ability to predict implant sizes, this can be limited due to varying availability of templating software and non-standardized imaging. In instances where certain implant sizes are not available, it is important for surgeons to be confident prior to proceeding that they will not require an unavailable implant size. This study has shown that the patients’ BMI cannot accurately predict the cup size required, but taller patients are more likely to require larger acetabular cup sizes. This is also reflected in the differences seen between genders. On average, men are taller than women and therefore are more likely to require larger acetabulum sizes compared to females. The information presented in this study can also help medical suppliers and manufacturers in predicting demands within the range of sizes they produce. By analysing the target population’s height, they will be able to predict the likely demands. For example, countries such as the Netherlands have a higher average height and are therefore more likely to consume large acetabular cup sizes.

Limitations to this study are mostly related to using acetabular cup size as a representation of overall acetabulum or bone size. Studies have shown a strong interobserver reliability in pre-operative templating sizes between surgeons [6]. The methodology used in this study would be more reliable if interobserver variation of cup size implanted intraoperatively had been studied, but this has not been done to date and would form the basis of further research. The authors of this study believe that large variations in the sizes that would be implanted in individual patients are unlikely due to little variation in techniques in implanting acetabular cups between surgeons. This study did not take into account the variety in pelvic anatomy between patients. This study presents results assessing the correlations with acetabular bone size, but we are unable to strongly recommend this to be an overall representation of the patient’s bone size. We believe that further studies assessing bone size ratios among patients would be of value. It is well known that women have different pelvic anatomy when compared to men, but partial Pearson’s correlation tests did not show a significant difference in the strength of correlation when using gender as a control for either BMI, height or weight. Information on the patients’ co-morbidities and social status were unavailable for analysis; however, the authors believe that this is unlikely to affect the overall finding of this study.

## Conclusions

Our study suggests that there is indeed a weakly positive linear association between BMI and cup size among total hip arthroplasty patients. This effect was, however, more significant for height and weight, and there was no significant association between obese and non-obese groups with small versus large cup size implanted. We therefore conclude that clinically there is no significant relationship between obesity and acetabular cup size and that the “big bones” claim maybe incorrect.
